# Supplementary material for: Genetic Relationships of 118 Castanea Specific Germplasms and Construction of Their Molecular ID Based on Morphological Characteristics and SSR Markers
Source: Plants (Basel). 2023 Mar 24;12(7):1438. doi: 10.3390/plants12071438 (PMC10096943; doi:10.3390/plants12071438)
Supplement: Supplementary file 1 [file plants-12-01438-s001.zip › Supplementary Table/Supplementary Table S2.docx]

**Table S2.** Ewens-Watterson Neutral Test.

| **Locus** | **Observed F** | **L95*** | **U95*** |
| --- | --- | --- | --- |
| P4 | 0.3895 | 0.2761 | 0.9049 |
| P82 | 0.2034 | 0.1504 | 0.5303 |
| P106 | 0.2610 | 0.1832 | 0.6675 |
| P108 | 0.2755 | 0.2014 | 0.7395 |
| P127 | 0.2747 | 0.2427 | 0.8804 |
| P138 | 0.2740 | 0.1591 | 0.5983 |

Note: Observed F: Observed sum of the squared of allelic frequency; L95*, U95*: The 95% confidence interval upper and lower limit.
