# Supplementary material for: Genetic Relationships of 118 Castanea Specific Germplasms and Construction of Their Molecular ID Based on Morphological Characteristics and SSR Markers
Source: Plants (Basel). 2023 Mar 24;12(7):1438. doi: 10.3390/plants12071438 (PMC10096943; doi:10.3390/plants12071438)
Supplement: Supplementary file 1 [file plants-12-01438-s001.zip › Supplementary Table/Supplementary Table S3.docx]

**Table S3.** Genetic diversity parameters of 112 Chinese chestnut materials.

| **Primer Name** | ***Na*** | ***NG*** | ***Ne*** | ***I*** | ***Ho*** | ***He*** | ***H*** | ***PIC*** | ***MAF*** |
| --- | --- | --- | --- | --- | --- | --- | --- | --- | --- |
| P4 | 5 | 8 | 2.5676 | 1.1774 | 0.1696 | 0.6133 | 0.6105 | 0.5622 | 0.5625 |
| P82 | 12 | 24 | 4.9163 | 1.8830 | 0.5536 | 0.8002 | 0.7966 | 0.7702 | 0.3125 |
| P106 | 9 | 17 | 3.8320 | 1.5526 | 0.9018 | 0.7424 | 0.7390 | 0.6969 | 0.3705 |
| P108 | 8 | 13 | 3.6296 | 1.5437 | 0.2321 | 0.7277 | 0.7245 | 0.6905 | 0.4464 |
| P127 | 6 | 12 | 3.6407 | 1.4210 | 0.6071 | 0.7286 | 0.7253 | 0.6753 | 0.3393 |
| P138 | 11 | 17 | 3.6502 | 1.5440 | 0.6161 | 0.7293 | 0.7260 | 0.6809 | 0.3795 |
| Mean | 8.5 | 15.1667 | 3.7061 | 1.5203 | 0.5134 | 0.7236 | 0.7203 | 0.6793 | 0.4018 |
