# Supplementary material for: Genetic Relationships of 118 Castanea Specific Germplasms and Construction of Their Molecular ID Based on Morphological Characteristics and SSR Markers
Source: Plants (Basel). 2023 Mar 24;12(7):1438. doi: 10.3390/plants12071438 (PMC10096943; doi:10.3390/plants12071438)
Supplement: Supplementary file 1 [file plants-12-01438-s001.zip › Supplementary Table/Supplementary Table S4.docx]

**Table S4.** List of 118 material numbers.

| **Code** | **Name of Variety** | **Germplasm Type** | **Species** | **Code** | **Name of Variety** | **Germplasm Type** | **Species** |
| --- | --- | --- | --- | --- | --- | --- | --- |
| 1 | Qing Zha | Variety (Department) | *Castanea mollissima* | 60 | Shen Ci Da Ban Li | Variety (Department) | *Castanea mollissima* |
| 2 | Shu He No.1 | Variety (Department) | *Castanea mollissima* | 61 | Xiao Jing Tie Li | Variety (Department) | *Castanea mollissima* |
| 3 | Shu He No.7 | Variety (Department) | *Castanea mollissima* | 62 | Zhong Chi Li | Variety (Department) | *Castanea mollissima* |
| 4 | Shu He No.10 | Variety (Department) | *Castanea mollissima* | 63 | Yue You No.9 | Variety (Department) | *Castanea mollissima* |
| 5 | Da Di Qing | Variety (Department) | *Castanea mollissima* | 64 | Te Zao | Variety (Department) | *Castanea mollissima* |
| 6 | Da Hong Pao | Variety (Department) | *Castanea mollissima* | 65 | Wu Mao Tie Li | Variety (Department) | *Castanea mollissima* |
| 7 | Da Gong Shu No.4 | Variety (Department) | *Castanea mollissima* | 66 | Duan Zhi Li | Variety (Department) | *Castanea mollissima* |
| 8 | You Zao No.1 | Variety (Department) | *Castanea mollissima* | 67 | Ban Li Zi | Variety (Department) | *Castanea mollissima* |
| 9 | Zao Li Zi | Variety (Department) | *Castanea mollissima* | 68 | Hong Ming Jian | Variety (Department) | *Castanea mollissima* |
| 10 | Jiu Jia Zhong | Variety (Department) | *Castanea mollissima* | 69 | Hu Bei You Li | Variety (Department) | *Castanea mollissima* |
| 11 | Jiao Zha | Variety (Department) | *Castanea mollissima* | 70 | Qing Mao Ruan Zha | Variety (Department) | *Castanea mollissima* |
| 12 | Jian Ding You Li | Variety (Department) | *Castanea mollissima* | 71 | CKD | Variety (Department) | *Castanea mollissima* |
| 13 | Dong Wang Ming Li | Variety (Department) | *Castanea mollissima* | 72 | DL-01 | Variety (Department) | *Castanea mollissima* |
| 14 | Hong Li | Variety (Department) | *Castanea mollissima* | 73 | DL-02 | Variety (Department) | *Castanea mollissima* |
| 15 | Xiao Luan Shi | Variety (Department) | *Castanea mollissima* | 74 | DL-03 | Variety (Department) | *Castanea mollissima* |
| 16 | Huang Qian Zhong Wan | Variety (Department) | *Castanea mollissima* | 75 | DL-04 | Variety (Department) | *Castanea mollissima* |
| 17 | Lian Hua Li | Variety (Department) | *Castanea mollissima* | 76 | MJH | Variety (Department) | *Castanea mollissima* |
| 18 | Yue You No.8 | Variety (Department) | *Castanea mollissima* | 77 | W4 | Variety (Department) | *Castanea mollissima* |
| 19 | Mi Feng Qiu | Variety (Department) | *Castanea mollissima* | 78 | W5 | Variety (Department) | *Castanea mollissima* |
| 20 | Wang Zi Tou No.7 | Variety (Department) | *Castanea mollissima* | 79 | XHC | Variety (Department) | *Castanea mollissima* |
| 21 | Gao Yuan No.1 | Variety (Department) | *Castanea mollissima* | 80 | XBC | Variety (Department) | *Castanea mollissima* |
| 22 | Shi Men Zao Shuo | Variety (Department) | *Castanea mollissima* | 81 | YBH | Variety (Department) | *Castanea mollissima* |
| 23 | Er Shui Zao | Variety (Department) | *Castanea mollissima* | 82 | YML | Variety (Department) | *Castanea mollissima* |
| 24 | Xin Zhuang No.2 | Variety (Department) | *Castanea mollissima* | 83 | Y46 | Variety (Department) | *Castanea mollissima* |
| 25 | Wei Hai Zao Shu | Variety (Department) | *Castanea mollissima* | 84 | Y47 | Variety (Department) | *Castanea mollissima* |
| 26 | Chu Shu Hong | Variety (Department) | *Castanea mollissima* | 85 | ZMZ | Variety (Department) | *Castanea mollissima* |
| 27 | Yan Hong | Variety (Department) | *Castanea mollissima* | 86 | ZA | Variety (Department) | *Castanea mollissima* |
| 28 | Xiao Xue | Variety (Department) | *Castanea mollissima* | 87 | No.6 | Variety (Department) | *Castanea mollissima* |
| 29 | Duan Zha | Variety (Department) | *Castanea mollissima* | 88 | No.9 | Variety (Department) | *Castanea mollissima* |
| 30 | Tai Shan Hong Li | Variety (Department) | *Castanea mollissima* | 89 | No.15 | Variety (Department) | *Castanea mollissima* |
| 31 | Gui Hua Xiang | Variety (Department) | *Castanea mollissima* | 90 | No.17 | Variety (Department) | *Castanea mollissima* |
| 32 | Yan Shan Zao Feng | Variety (Department) | *Castanea mollissima* | 91 | No.18 | Variety (Department) | *Castanea mollissima* |
| 33 | Yang Guang No.2 | Variety (Department) | *Castanea mollissima* | 92 | No.101 | Variety (Department) | *Castanea mollissima* |
| 34 | Bo Ke Chi Li | Variety (Department) | *Castanea mollissima* | 93 | 102B | Variety (Department) | *Castanea mollissima* |
| 35 | Huang Li Pu | Variety (Department) | *Castanea mollissima* | 94 | No.105 | Variety (Department) | *Castanea mollissima* |
| 36 | Mao Pu | Variety (Department) | *Castanea mollissima* | 95 | No.108 | Variety (Department) | *Castanea mollissima* |
| 37 | Xin Yi You Li | Variety (Department) | *Castanea mollissima* | 96 | No.203 | Variety (Department) | *Castanea mollissima* |
| 38 | Chui Zhi Li | Variety (Department) | *Castanea mollissima* | 97 | No.207 | Variety (Department) | *Castanea mollissima* |
| 39 | Nian Di Ban | Variety (Department) | *Castanea mollissima* | 98 | No.213 | Variety (Department) | *Castanea mollissima* |
| 40 | Yue Xi No.2 | Variety (Department) | *Castanea mollissima* | 99 | No.302 | Variety (Department) | *Castanea mollissima* |
| 41 | Wu Ke Li | Variety (Department) | *Castanea mollissima* | 100 | No.1059 | Variety (Department) | *Castanea mollissima* |
| 42 | Jie Jie Hong | Variety (Department) | *Castanea mollissima* | 101 | No.1061 | Variety (Department) | *Castanea mollissima* |
| 43 | Cen Kou Da Li | Variety (Department) | *Castanea mollissima* | 102 | No.1504 | Variety (Department) | *Castanea mollissima* |
| 44 | Liu Yue Bao | Variety (Department) | *Castanea mollissima* | 103 | 8017 | Variety (Department) | *Castanea mollissima* |
| 45 | Ba Yue Hong | Variety (Department) | *Castanea mollissima* | 104 | Liu He Hong Li | wild | *Castanea mollissima* |
| 46 | Hua Gai | Variety (Department) | *Castanea mollissima* | 105 | － | wild | *Castanea mollissima* |
| 47 | Huang Qian Wu Hua | Variety (Department) | *Castanea mollissima* | 106 | － | wild | *Castanea mollissima* |
| 48 | Su Cheng Da Li | Variety (Department) | *Castanea mollissima* | 107 | － | wild | *Castanea mollissima* |
| 49 | Da You Li | Variety (Department) | *Castanea mollissima* | 108 | － | wild | *Castanea mollissima* |
| 50 | Gui Xuan 72-1 | Variety (Department) | *Castanea mollissima* | 109 | － | wild | *Castanea mollissima* |
| 51 | Long An No.1 | Variety (Department) | *Castanea mollissima* | 110 | Gan Yu No.1 | wild | *Castanea mollissima* |
| 52 | He Bei Zun Yu | Variety (Department) | *Castanea mollissima* | 111 | － | wild | *Castanea mollissima* |
| 53 | Kui Li | Variety (Department) | *Castanea mollissima* | 112 | － | wild | *Castanea mollissima* |
| 54 | Jiu Yue Han | Variety (Department) | *Castanea mollissima* | 113 | Yin Ji | wild | *Castanea crenata* |
| 55 | Mei Gui Hong | Variety (Department) | *Castanea mollissima* | 114 | － | wild | *Castanea crenata* |
| 56 | Wang Jie Gen | Variety (Department) | *Castanea mollissima* | 115 | － | wild | *Castanea crenata* |
| 57 | Chen Guo You Li | Variety (Department) | *Castanea mollissima* | 116 | － | wild | *Castanea crenata* |
| 58 | Shuang He Da Hong Pao | Variety (Department) | *Castanea mollissima* | 117 | － | wild | *Castanea henryi* |
| 59 | Er Xin Zao | Variety (Department) | *Castanea mollissima* | 118 | － | wild | *Castanea sativa* |

Note: ‘－’ represent the material is wild resource, does not have variety name.
